# Osteoid osteoma of the foot: Presentation, treatment and outcome of a multicentre cohort

**DOI:** 10.1007/s00508-021-01966-0

**Published:** 2021-11-04

**Authors:** Maria A. Smolle, Magdalena M. Gilg, Felix Machacek, Miroslav Smerdelj, Per-Ulf Tunn, Blaz Mavcic, Nenad Lujic, Jelena Sopta, Lauris Repsa, Jasminka Igrec, Andreas Leithner, Marko Bergovec

**Affiliations:** 1 Department of Orthopaedics and Trauma, Medical University of Graz, Auenbruggerplatz 5, 8036 Graz, Austria; 2 Department of Orthopaedic Surgery, Orthopaedic Hospital Gersthof, Vienna, Austria; 3 Department of Orthopaedic Surgery, Clinical Hospital Centre Zagreb, Zagreb, Croatia; 4 Department of Tumour Orthopaedics, HELIOS Klinikum Berlin-Buch, Berlin-Buch, Germany; 5 Department of Orthopaedic Surgery, Faculty of Medicine, University Medical Centre Ljubljana, Ljubljana, Slovenia; 6 Institute for Orthopedic Surgery Banjica, Medical Faculty, University of Belgrade, Belgrade, Serbia; 7 Department of Orthopaedics, Riga Stradins University, Riga, Latvia; 8 Department of Radiology, Medical University of Graz, Graz, Austria

**Keywords:** Osteoid osteoma, Foot tumour, Diagnosis, Symptoms, Radiology

## Abstract

**Background:**

Osteoid osteomas of the foot are rare, with a varying and atypical clinical as well as radiological presentation impeding early diagnosis and treatment. The aim of the present multicentre study was to 1) analyze epidemiological, clinical and radiological findings of patients with foot osteoid osteomas and to 2) deduce a diagnostic algorithm based on the findings.

**Methods:**

A total of 37 patients (25 males, 67.6%, mean age 23.9 years, range 8–57 years) with osteoid osteomas of the foot were retrospectively included, treated between 2000 and 2014 at 6 participating tertiary tumor centres. Radiographic images were analyzed, as were patients’ minor and major complaints, pain relief and recurrence.

**Results:**

Most osteoid osteomas were located in the midfoot (*n* = 16) and hindfoot (*n* = 14). Painful lesions were present in all but one patient (97.3%). Symptom duration was similar for hindfoot and midfoot/forefoot (*p* = 0.331). Cortical lesions required fewer x‑rays for diagnosis than lesions at other sites (*p* = 0.026). A typical nidus could be detected in only 23/37 of x‑rays (62.2%), compared to 25/29 CT scans (86.2%) and 11/22 MRIs (50%). Aspirin test was positive in 18/20 patients (90%), 31 patients (83.8%) underwent open surgery. Pain relief was achieved in 34/36 patients (outcome unknown in one), whilst pain persisted in two patients with later confirmed recurrence.

**Conclusions:**

As previously reported, CT scans seem to be superior to MRIs towards detection of the typical nidus in foot osteoid osteomas. In patients with unclear pain of the foot and inconclusive x‑rays, osteoid osteoma should be considered as differential diagnosis.

## Introduction

Osteoid osteomas constitute benign bone tumors with males being affected three times more often than females [1]. The typical clinical presentation consists of pain—especially at night—that is promptly but only for a short time relieved by salicylates and other nonsteroidal anti-inflammatory drugs [2]. Moreover, local tenderness and swelling may be present, potentially caused by the hypervascularity of the tumour itself and the production of prostaglandins [3]. At radiography, an intracortical nidus with a strongly mineralized centre and accompanying cortical thickening and sclerosis may be found [4]. Depending on the location of the nidus on radiograph, three types can be distinguished: cortical, medullary/cancellous and subperiosteal [5]. Their clinical presentation may be different from conventional osteoid osteomas, including symptoms resembling arthritis, such as joint swelling, stiffness, tenderness and effusion [3, 6]. Osteoid osteomas of the foot often cause a diagnostic dilemma, due to their rarity, varying and atypical symptoms and uncommon radiological appearance [7, 8]. They account for 19.4% of all benign bone tumours in this region, with a particular predilection for talus and calcaneus [9].

Due to their rarity, there are only few case series on osteoid osteomas of the foot region, with limited patient numbers [10]. Over a 20-year period, Houdek et al. were able to include 13 patients with foot and ankle osteoid osteomas [11]. In the most recent study on osteoid osteomas of the foot and ankle region, Gurkan et al. reported on 9 patients [7]. In a systematic review by Jordan et al., 68.1% of included publications were case reports [12]. In this field, no multicentre study has been published yet on a group of over 30 patients with a uniform observational study protocol.

The aim of the present study was to 1) analyze the epidemiological, clinical and radiological findings of 37 patients with osteoid osteomas of the foot, and to 2) propose a diagnostic algorithm based on our observations. We hypothesized that specific symptoms, imaging modalities and clinical tests may be indicative of osteoid osteomas of the foot.

## Patients, material and methods

Altogether, 37 patients with osteoid osteoma of the foot, treated between 2000 and 2014 at 6 participating tertiary tumour centres, were retrospectively included. Patients were selected from prospectively maintained tumour databases at the respective centres, using either histological or (in the case no histological analysis had been performed) unambiguous radiological diagnosis of osteoid osteoma of the foot region [13]. Thus, patients were excluded if preoperative imaging had been inconclusive and the suspected diagnosis had not been confirmed histologically. Of all patients, 25 were male (67.6%) and 12 female (32.4%). The mean age of patients at diagnosis of disease was 23.9 years (8–57 years). Images (i.e. X‑ray, computed tomography, CT, magnetic resonance imaging, MRI, bone scan) performed during the diagnostic process were analyzed for the presence of a typical nidus, being a radiolucent area and with or without surrounding sclerosis [12]. Furthermore, symptom duration prior to diagnosis, patients’ minor and major complaints and primarily assumed differential diagnoses were ascertained using medical reports. Anatomical location of the lesion was subdivided into hindfoot (i.e. calcaneus, talus), midfoot (i.e. metatarsals, cuboid, cuneiform, navicular), and forefoot (i.e. phalanges). Ethical approval for this retrospective study had been obtained at the involved centres. The study was performed according to the Declaration of Helsinki.

### CT and MRI protocols

Thin-section axial multiplanar reformatted CT images in three anatomical planes were obtained with a bone algorithm and viewed with bone window setting in order to detect the nidus, identify the origin of a tumor and differentiate osteoid osteoma from other sclerotic lesions, such as arthritis, stress fracture, enostosis, osteoblastoma or osteomyelitis.

As recommended by the ESSR [14] for bone tumors, MRI should include standard T1-weighted and T2-weighted spin-echo images with optional use of fat saturation and intravenous application of gadolinium chelates in order to assess the intralesional matrix characteristics, cortical destruction, soft tissue extension, joint and/or neurovascular invasion. The field strength of the MRI scanner should be at least 1 T, but preferentially 1.5 or 3 T. In order to cover the entire lesion and surrounding normal tissues, axial sequences with high spatial resolution were used after optimization of the matrix to achieve high-plane spatial resolution and high signal-to-noise ratio in a reasonable examination time. In our study, all gathered MRI examinations from the respective centres fulfilled the basic examination requirements whereby minimum MRI protocol included: (1) coronal STIR sequence with small FOV covering two joints, (2) axial spin echo T1-weighted sequence (without fat saturation), axial fast/turbo spin echo T2-weighted sequence (without fat saturation) with identical slice thickness and coverage as for the T1-weighted sequence.

### Treatment

Patients either underwent curettage of the lesion or radiofrequency ablation (RFA) under spinal or general anesthesia. During surgery, preparation of skin and subcutaneous structures was followed by resection of the lesion using curettes and high-speed burrs either through a small cortical window (if intramedullary) or on the bony cortex itself (if cortical).

Upon RFA, contiguous CT scans using stereotactic navigation systems (e.g. CAS-One, CAScination, Bern, Switzerland) were initially carried out to localize the nidus. Subsequently, the nidus was opened with a biopsy needle or coaxial drill system and the active tip of the monopolar RFA-electrode inserted [15]. The tip of the electrode was heated up to 90 °C for 6 min, resulting in a spherical (5 mm electrode) or cylindrical (8 mm electrode) zone. In the case of large tumours or electrode placement off-centre, this procedure was repeated with relocated electrodes in order to cover the entire lesion. A postprocedural CT scan was performed to confirm lack of soft tissue edema or hematoma.

### Outcome

Treatment and outcome variables pain relief (with preoperative and postoperative visual analogue scale (VAS) scores, where available), local recurrence, postoperative mobility and complications where likewise recorded. According to the European Society of Musculoskeletal Radiology (ESSR) recommendation, patients were followed-up at least once following RFA/surgery or until cessation of symptoms [14].

### Statistical analysis

Statistical analysis was performed using Stata version 15.1 (StataCorp, Texas, US), χ^2^-testsand t‑tests were performed to analyze differences between groups. A two-sided *p*-value of < 0.05 was considered statistically significant.

## Results

### Demographic features

**Fig. 1:**
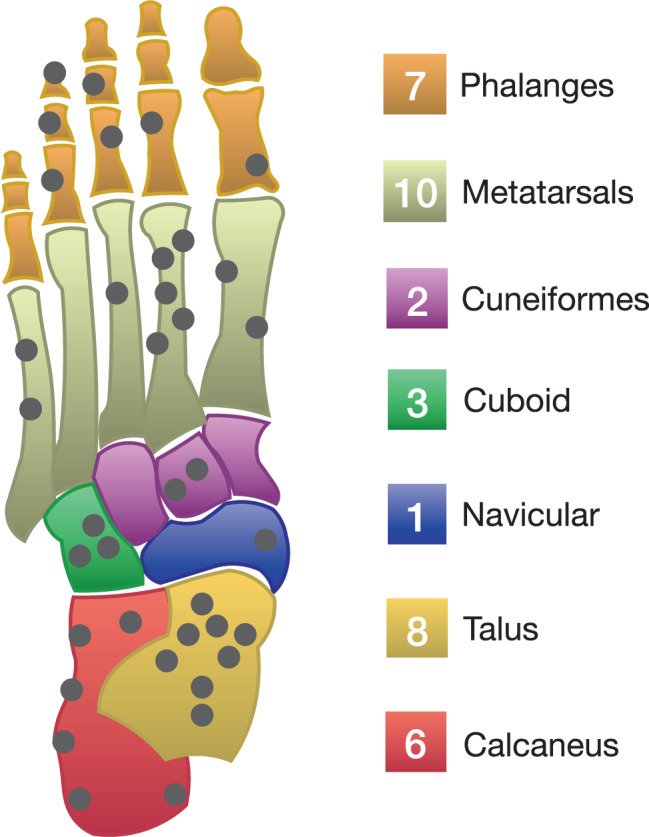
Location of the osteoid osteomas in the foot as observed in the present study (*n* = 37)

**Fig. 2:**
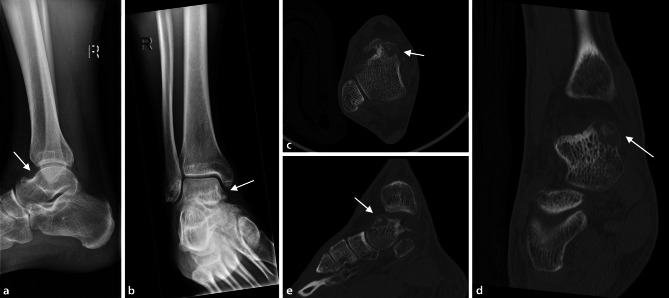
**a**, **b** 18-year-old male patient with osteoid osteoma located in the talar neck of the right foot adjacent to the medial talar shoulder, presenting as blurred, lucent region on the anteroposterior (ap) (**a**) and lateral (**b**) view (*arrows* pointing at the regions of interest). **c**–**e** Transverse (**c**), frontal (**d**) and sagittal (**e**) CT scans showing osteolytic lesion with cortical thinning and central sclerosis in the medial aspect of the talar neck (*arrows* pointing at the regions of interest)

**Fig. 3:**
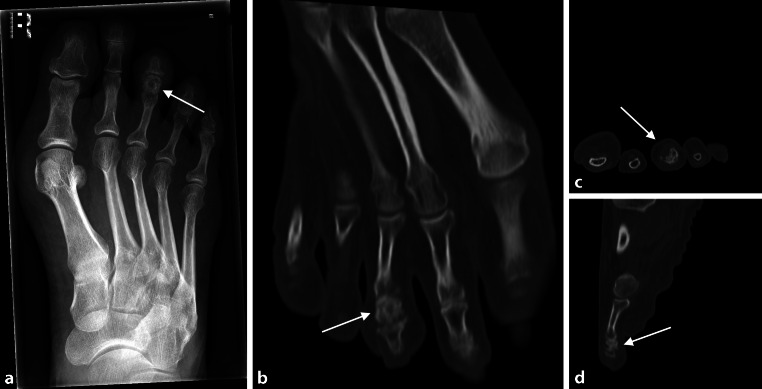
**a** 57-year-old female patient with osteoid osteoma of the right foot 3rd middle phalanx. X‑ray with cortical thickening and central osteolytic area occupying the entire middle phalanx of the 3rd ray (*arrows* pointing at the regions of interest). **b**–**d** Transverse (**b**), frontal (**c**), and sagittal (**d**) CT scans with expansive process of the distended middle phalanx, partly sclerotic, partly lytic with sporadic cortical destruction and surrounding soft tissue swelling (*arrows* pointing at the regions of interest)

**Fig. 4:**
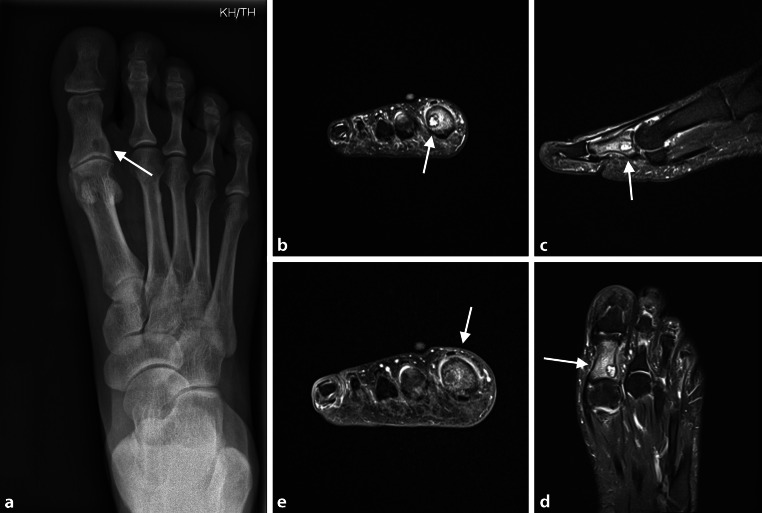
**a** 55-year-old female patient with osteoid osteoma of the 1st proximal phalanx of the right foot. X‑ray with lytic lesion and cortical thickening at the medial aspect of the base of the 1st proximal phalanx (*arrows* pointing at the regions of interest). **b**–**e** MRI scans showing well-circumscribed sclerotic lesion of 6 mm in size with extensive bone edema. Minor synovial reactive effusion at the 1st metatarsophalangeal joint (**b** T2 Turbo spin echo (TSE) fatsat (FS); **c** ,**d** T2 FS; **e** T1 TSE + Contrast Agent; arrows pointing at the regions of interest)

Of the osteoid osteomas 16 were located in midfoot (43.2%), 14 in the hindfoot (37.8%; Fig. 1, Fig. 2), and 7 in the forefoot (18.9%; Fig. 3. and Fig. 4.). The majority of osteoid osteomas had a cortical location (*n* = 17; 45.9%), followed by an intramedullary position (*n* = 7; 18.9%).

Median symptom duration prior to correct diagnosis was 12 months (interquartile range, IQR: 6–24 months). There was no difference in duration of symptoms depending on the location of the lesion with the numbers available (hindfoot vs. midfoot/forefoot; t‑test *p* *=* 0.331). The most prevalent major symptom was a painful mass in all but one patient, who presented with a swelling only (Table 1). Accompanying symptoms included nocturnal pain (*n* = 18; 48.6%), swelling (*n* = 15; 40.5%) and local tenderness (*n* = 15; 40.5%). Further accompanying symptoms are listed in Table 1.

**Table 1:** General patient-related and tumor-related features

			Frequency	%	Missing
**Centres**	*A*		7	18.9	0
	*B*		5	13.5	
	*C*		7	18.9	
	*D*		9	24.4	
	*E*		2	5.4	
	*F*		7	18.9	
**Gender**	*Male*		25	67.6	0
	*Female*		12	32.4	
**Location**	***Hindfoot***	*Talus*	8	21.6	0
		*Calcaneus*	6	16.2	
	***Midfoot***	*Cuneiform*	2	5.4	
		*Cuboid*	3	8.1	
		*Navicular*	1	2.7	
		*Metatarsal*	10	27.0	
	***Forefoot***	*Phalanx*	7	18.9	
**Position in bone**	*Cortical*		17	45.9	6
	*Intramedullary*		7	18.9	
	*Juxta-articular*		5	13.5	
	*Other*		2	5.4	
**Major symptoms**	*Pain/tenderness*		31	83.3	0
	*Swelling*		1	2.7	
	*Pain* *+* *swelling*		5	13.5	
**Minor Symptoms**	*Night pain*	*No*	19	51.4	0
		*Yes*	18	48.6	
	*Swelling*	*No*	22	59.5	0
		*Yes*	15	40.5	
	*Pain on weight bearing*	*No*	30	81.1	0
		*Yes*	7	18.9	
	*Synovitis*	*No*	33	89.2	0
		*Yes*	4	10.8	
	*Restricted motion*	*No*	32	86.5	0
		*Yes*	5	13.5	
	*Tenderness*	*No*	22	59.5	0
		*Yes*	15	40.5	
	*Stiffness*	*No*	33	89.2	0
		*Yes*	4	10.8	

### Diagnostic pathway

In all patients, at least one radiograph was performed during the diagnostic pathway (mean: 2; range: 1–5); however, the typical nidus surrounded by a distinct zone of sclerosis could only be detected in 23 cases (62.2%). Significantly more radiographs became necessary in osteoid osteomas located elsewhere than cortical (2.6 vs. 1.9 images; t‑test *p* *=* 0.026). There was no difference whether a nidus could be detected on x‑rays depending on cortical (64.7% with nidus) vs. all other locations (57.1% with nidus; χ^2^-test; *p* = 0.667).

In 29 of the 37 patients a CT scan was performed with a nidus visible in 25 images (86.2%). Furthermore, MRI scans were performed in 22 patients and a nidus was detectable in 11 cases (50%). In 19 patients, a bone scan was performed, being positive in all 19 cases but overall being considered highly unspecific. Altogether, each patient had a mean of 4 imaging studies (range: 2–9; either X‑ray, CT, MRI or bone scan) before the correct diagnosis was reached.

Differential diagnoses prior to correct diagnosis included osteomyelitis and tumor (i.e. giant cell tumor [*n* = 1], Ewing sarcoma [*n* = 1], osteoblastoma [*n* = 1], enchondroma [*n* = 1], non-ossifying fibroma [*n* = 1]) in 5 cases each, a posttraumatic condition in 4 and bone edema in 2 cases. Complex regional pain syndrome, tenosynovitis and plantar fasciitis were further differential diagnoses in three cases. In 17 cases (45.9%), no working diagnosis had been reached before osteoid osteoma was confirmed by histology (typically circumscribed nodule of woven bone and osteoid, prominent osteoblastic rimming, surrounded by cortical/trabecular bone and fibrovascular tissue [16]).

An aspirin-test (i.e. acetylsalicylic acid 500 mg twice daily for 2–5 days) was performed in 20 patients (54.1%) prior to invasive treatment to further strengthen the suspected diagnosis, with the test being positive (i.e. pain relief upon aspirin intake) in 18 individuals (90%).

### Treatment

Of the patients 31 (83.8%) underwent curettage of the lesion as definitive surgery. In one of these patients, phenol had been used as an adjuvant. Further four patients (10.8%) were treated with RFA and one patient (2.7%) with curettage + RFA (two procedures). In another case (2.7%), where the tumour was located juxta-articular in the talar region, arthroscopic removal of the nidus was performed. In 31 of 37 patients (83.8%), histological specimens were obtained confirming the diagnosis of osteoid osteoma. In one patient with arthroscopic nidus removal, in one with curettage and in those four with RFA only, no histological specimens had been analyzed.

### Outcome

In 34 of 36 patients (94.4%), pain relief following RFA or surgery could be achieved, whilst in 1 patient (2.8%), this outcome measure was unknown. In those 9 patients with VAS scores available, median preoperative and postoperative VAS scores were 4 (IQR: 4–5) and 0 (IQR: 0–0), respectively.

In two patients with prior curettage, complaints persisted due to incomplete tumour removal (one reporting on pain in extreme positions of the foot and the other one with recurrent pain upon weight bearing), confirmed radiologically as “recurrence”, requiring further surgical procedures. Furthermore, at the final examination, 14 patients were not restricted in daily activities following surgery (37.8%), 19 patients were fully-weight bearing (without information on potential complaints or restrictions in daily activities [51.4%]), one patient reported occasional pain during excessive physical activities (2.7%) and one patient noticed reduced range of motion in the subtalar joint following curettage of a cortical osteoid osteoma in the calcaneus (2.7%). No surgical or RFA-related complications were observed.

### Diagnostic algorithm

**Fig. 5:**
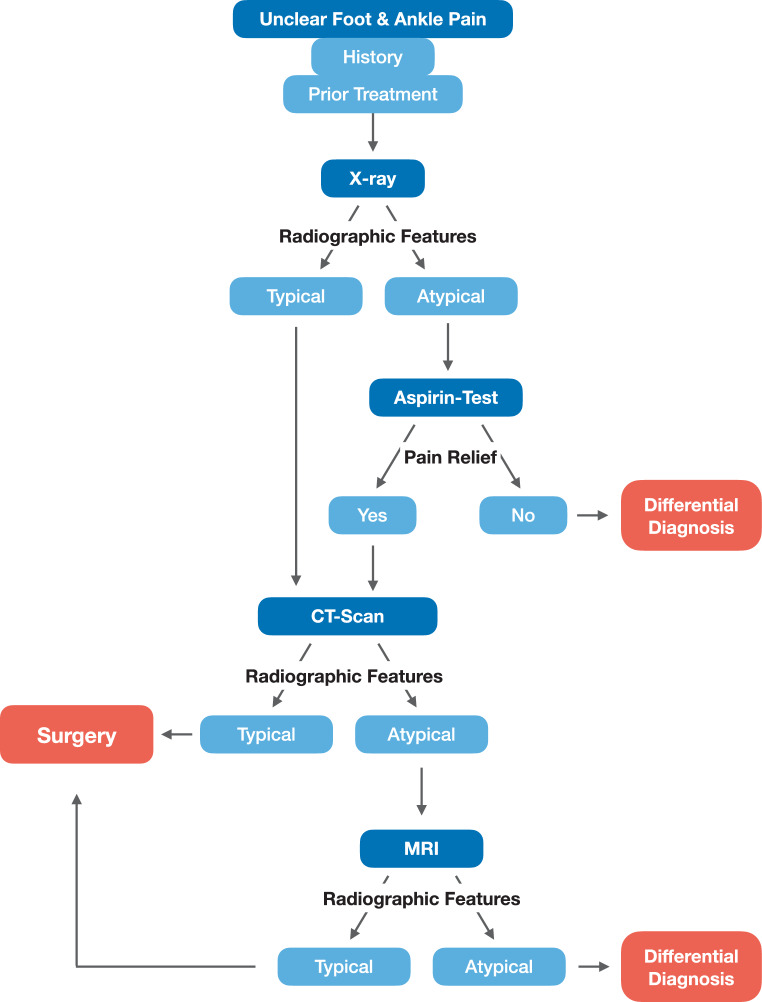
Algorithm to aid diagnosis of foot osteoid osteomas

Based on the observations of the present study, we deduced a diagnostic algorithm (Fig. 5). An integral part of this algorithm is the early introduction of an aspirin test, consisting of aspirin 500 mg twice daily for 2–5 subsequent days. Should this be positive, a CT scan is recommended to visualize the nidus. Only after a negative CT scan, MRI scans may be performed for differential diagnosis.

## Discussion

According to the present study, the often atypical symptoms of foot osteoid osteomas cause a diagnostic delay. The aspirin-test—if performed—is positive in almost all cases and should be initiated early in the diagnostic process. Furthermore, CT scans are superior to MRI in detecting the typical nidus, thus aiding early diagnosis.

In our study, the median delay from onset of symptoms to diagnosis was 1 year, which is less than the median of 1.8 years observed in a systematic review on foot and ankle osteoid osteomas [12]. This may be explained by the fact that our study covered a relatively recent time period where imaging modalities were already quite accurate and that we included only patients treated at tertiary orthopedic tumor centres with a high expertise.

Yet, a mean of 4 imaging studies were necessary to reach the correct diagnosis. A nidus could be detected equally frequent on x‑rays irrespective of the location of osteoid osteomas within bone (i.e. cortical vs. non-cortical). Therefore, even at highly specialized centres, osteoid osteomas of the foot are difficult to diagnose. As reported previously, CT scans were superior to MRI in order to reach the diagnosis of osteoid osteoma [12]. In our study, the typical nidus was visible in 86.2% of cases, which is comparable to the 96.4% reported by Jordan et al. in their systematic review including 223 cases of osteoid osteomas located in the foot and ankle region [12]. On the other hand, MRI revealed a nidus in half of our cases only, due to the fact that in small lesions the nidus and surrounding cortex often show identical signal intensity [17]. Thus, unsurprisingly, a misdiagnosis rate of 35% has been previously reported when MRI was used as the primary imaging modality [12, 18]. In patients with non-cortical osteoid osteomas, significantly more X‑rays were performed during the diagnostic pathway, which could be related to their atypical location and configuration, complicating detection and diagnosis. In our cohort, the most common differential diagnoses were malignant tumors other than osteoid osteoma and osteomyelitis, probably caused by the edematous zones visible on MRI and symptoms as swelling and local tenderness [8, 14]. For example, in the case report by Thiermann et al., an osteoid osteoma of the distal phalanx of the great toe was initially wrongly diagnosed and treated as an ingrown nail with concomitant osteomyelitis, due to bone and soft tissue edema as well as contrast enhancement detected on MRI [8]. These features may also wrongly lead to the differential diagnosis of trauma, which was the case in 10.8% of our patients. It was not assessed to which extent the differential diagnoses were of help in reaching the final diagnosis, although it is more likely that especially the diagnosis of trauma may have resulted in an unnecessary diagnostic delay. The aspirin test, i.e. administration of aspirin with subsequent pain relief, was positive in 90% of our patients prior to invasive treatment, with the number being relatively higher than previously reported 72% [12].

The majority of patients underwent surgical resection of the osteoid osteoma, which is in line with previous observations [7, 12]; however, thermal ablation using, e.g. RFA, is becoming more common [19]. Nevertheless, due to critical structures in close vicinity to bone in the foot, as well as the risk for skin burns and necrosis following RFA [20, 21], open surgery may be favored. Consistent with other reports, in over 90% of our patients symptom relief could be achieved by either RFA or open surgery [22, 23]. Thereafter, as recommended by ESSR, at least one follow-up appointment should be scheduled until patients are free of complaints [14].

Based on our results, the following diagnostic approach for patients with unclear foot pain is suggested: radiographs should be followed by an aspirin test. Upon pain relief, a CT scan of the affected region should be performed in order to visualize the pathognomic nidus.

Limitations of the present study include the non-uniform assessment of patients as they had been treated at different tertiary tumor centres. Furthermore, due to the retrospective design of the study, some data—and in particular regarding follow-up time—was impossible to ascertain; however, with the main aim being to perform a thorough analysis of clinical, diagnostic and treatment-related parameters in patients with osteoid osteoma of the foot, rather than analyzing any outcome variables in the long term, the lack of follow-up information has not affected the study’s research question.

## Conclusion

The present retrospective multicentre study confirmed previously published results on osteoid osteomas of the foot region. Potentially due to treatment in highly specialized tertiary tumor centres, the diagnostic delay was relatively shorter than previously described. CT scans proved to be highly sensitive for detection of foot osteoid osteomas, whilst MRI—at least in the present study—was less reliable. An aspirin test is recommended for clinicians in practice prior to CT or MRI examinations in order to strengthen the suspected diagnosis.
